# Rotational spectroscopic study of S-methyl thioformate: A global laboratory analysis of ground and excited torsional states up to 660 GHz*

**DOI:** 10.1051/0004-6361/202038200

**Published:** 2020-12-07

**Authors:** A. Jabri, B. Tercero, L. Margulès, R. A. Motiyenko, E. A. Alekseev, I. Kleiner, J. Cernicharo, J-C. Guillemin

**Affiliations:** 1 Laboratoire Interuniversitaire des Systèmes Atmosphériques (LISA), UMR CNRS 7583, Université Paris-Est-Créteil, Université de Paris, Institut Pierre Simon Laplace (IPSL), 61 av. du Général de Gaulle, 94010 Créteil, France; 2 Observatorio Astronómico Nacional (OAN-IGN). Calle Alfonso XII, 3, 28014 Madrid, Spain; 3 Observatorio de Yebes (IGN). Cerro de la Palera s/n, 19141 Yebes, Guadalajara, Spain; 4 Univ. Lille, CNRS, UMR 8523 - PhLAM - Physique des Lasers Atomes et Molécules, 59000 Lille, France; 5 Radiospectrometry Department, Institute of Radio Astronomy of NASU, Kharkov, Ukraine; 6 Instituto de Física Fundamental (IFF-CSIC). Calle Serrano 123, 28006 Madrid, Spain; 7 Univ. Rennes, Ecole Nationale Supérieure de Chimie de Rennes, CNRS, ISCR-UMR 6226, 35000 Rennes, France

**Keywords:** line: identification, catalogs, ISM: abundances, submillimeter: ISM, methods: laboratory: molecular

## Abstract

**Context:**

S-methyl thioformate CH_3_SC(O)H is a monosulfur derivative of methyl formate, a relatively abundant component of the interstellar medium (ISM). S-methyl thioformate being, thermodynamically, the most stable isomer, it can be reasonably proposed for detection in the ISM.

**Aims:**

This work aims to experimentally study and theoretically analyze the ground and first torsional excited states for CH_3_SC(O)H in a large spectral range for astrophysical use.

**Methods:**

S-methyl thioformate was synthesized as a result of a reaction of methyl mercaptan with acetic-formic anhydride. The millimeter-wave spectrum was then recorded for the first time from 150 to 660 GHz with the solid-state spectrometer located at Lille.

**Results:**

A set of 3545 lines is determined and combined with 54 previously measured lines in the microwave region, belonging to ground state *ν*_t_ = 0 as well as 1391 transitions in the first excited state of torsion *ν*_18_ = 1. Some 164 lines were also assigned to *ν*_18_ = 2 for the A-species. A global fit was performed using the BELGI-C_s_ code taking into account the large splitting of A and E lines due to methyl internal rotation motion with a relatively low barrier, V_3_ = 127.4846(15) cm^–1^.

**Conclusions:**

Using our spectroscopy work, a deep search of S-methyl thioformate was carried out in the IRAM 30m and ALMA data of different high-mass star-forming regions (Orion KL and Sgr B2). We derived an upper limit to the CH_3_SC(O)H column density in these regions.

## 1 Introduction

In recent years, the detection of many complex organic molecules (COMs) in the interstellar medium (ISM) and circumstellar envelopes has been made possible by substantial progress accomplished in astrophysical observation. According to the Cologne Database for Molecular Spectroscopy (CDMS, Endres et al. 2016) and to the recent review of McGuire (2018), around 204 different molecules have been detected, and almost a third of them contain more than five atoms (Herbst & Van Dishoeck 2009). Most of the molecular identifications were accomplished by recording their spectra in the laboratory and comparing them to the interstellar surveys by means of microwave, millimeter- or submillimeter-wave telescopes.

The detection of molecular species in space by microwave, millimeter- and submillimeter-wave telescopes would have been impossible without dedicated studies in the laboratory addressing the high-resolution rotational and rovibrational spectroscopy, both in theory and experimentally, of relevant species. Precise knowledge of the rotational spectra was the key point for the detections of many complex molecules compiled and updated in the existing international databases such as CDMS (Endres et al. 2016), Splatalogue, JPL catalog (Pickett et al. 1998), NIST catalog (Lovas 2004), and Toyama Microwave Atlas for spectroscopists and astronomers. To gain this knowledge, a common strategy is to combine experimental microwave, millimeterand submillimeter-wave spectroscopy, followed by the analysis of these spectra using appropriate effective quantum mechanical Hamiltonians and ab initio calculations, if no information on the rotational constants is available from previous studies.

In the last decades, several molecules exhibiting internal rotations of the methyl CH_3_ group have been detected in the interstellar and circumstellar media; for example, methanol (CH_3_OH, Ball et al. 1970), methyl formate and its isotopologues (HCOOCH_3_, Churchwell & Winnewisser 1975; Demyk et al. 2008; Favre et al. 2014; Carvajal et al. 2009; Haykal et al. 2014; Kobayashi et al. 2013), acetaldehyde (CH_3_CHO, Fourikis et al. 1974), acetic acid (CH_3_COOH, Mehringer et al. 1997; Remijan et al. 2003), and acetamide (CH_3_CONH_2_, Hollis et al. 2006).

Sulfur-bearing molecules are important species when it comes to understanding the chemical evolution of hot cores, and their molecular ratios have previously been used as chemical clocks to obtain information about the age of those hot cores (Wakelam et al. 2004; Charnley 1997). However, the chemistry of interstellar sulfur is still not well understood. The observed abundance of sulfur-bearing species in dense clouds only represents about 0.1% of the abundance of sulfur in diffuse clouds (where it corresponds almost to its solar abundance). There is still some mystery surrounding this missing sulfur and what its reservoir would be (Anderson et al. 2013). About 20 different molecules containing sulfur have been identified in space so far, and among them, only methyl mercaptan (CH_3_SH) exhibits observable internal rotation splitting (called A-E splitting) (Linke et al. 1979; Xu et al. 2012; Kolesniková et al. 2014; Zakharenko et al. 2019).

S-methyl thioformate CH_3_SC(O)H is the sulfur analog of methyl formate CH_3_OC(O)H, a relatively abundant and ubiquitous molecule in the ISM (Brown et al. 1975; Churchwell & Winnewisser 1975). It is also detected in the comet Hale-Bopp (Bockelée-Morvan et al. 2000) and in low-mass star-forming regions (hot corinos) such as NGC 1333-IRAS4B and IRAS2A (Bottinelli et al. 2007). Methyl thioformate can be thus considered as a potentially detectable species in this medium because most of the abundant oxygen derivatives have the corresponding sulfur derivative detected in the ISM (Cernicharo et al. 1987).

Relatively few laboratory spectroscopic studies exist on internal rotors containing sulfur, and in the case of methyl thioformate those studies mainly concern transitions in the ground torsional state *ν*_t_ = 0. Two isomers exist for methyl thioformate where either the sp^3^ oxygen atom of methyl formate HCOOCH_3_ is substituted by a sulfur atom (S-methyl thioformate CH_3_SC(O)H) or the sp^2^ oxygen is substituted (O-methyl thioformate CH_3_OC(S)H). In 2014 Senent et al. (2014) performed highlevel quantum chemical calculations to determine the spectro-scopic parameters (rotational and centrifugal distortion constants as well as the potential barrier hindering the internal rotation of the methyl group). They also characterized the relative energies for the two isomers, CH_3_SC(O)H and CH_3_OC(S)H. Figure 2 of Senent et al. (2014) shows the two isomers with their possible conformers, cis- and trans-, depending on the position of the methyl group relative to that of the C = O or C = S bonds for CH_3_SC(O)H and CH_3_OC(S)H, respectively. The cis- S-methyl thioformate CH_3_SC(O)H is determined as the most stable structure at 1134 cm^–1^ below the trans- CH_3_SC(O)H, and at 4372.2 and 6335.8 cm^–1^ below the cis- and trans- configurations of O-methyl thioformate CH_3_OC(S)H, respectively. In the present paper, we focus on the spectroscopy of the cis- S-methyl thioformate CH_3_SC(O)H as it corresponds to the most stable species.

To our knowledge, only two spectroscopic studies are available for CH_3_SC(O)H: Jones et al. (1976) recorded the microwave spectrum between 10 and 41 GHz, as well as an infrared spectrum recorded at low resolution between 50 and 4000 cm^–1^. Although they measured about 40 lines of the torsional A-species in the R-branch (corresponding to the a-type selection rule) and in the R- and Q-branches (corresponding to the b-type selection rule), their search for the A-E splitting due to internal rotation was unsuccessful because of the poor information about the barrier height to internal rotation V_3_. Self consistent field (SCF) ab initio calculations allowed them to conclude that their measured lines correspond to the cis- CH_3_SC(O)H isomer. Using Stark-effect measurements, they were able to determine the electric dipole moment values μ_a_ and μ_b_ in the principal axis system to be 1.52 and 0.43 Debye, respectively. Later on, Caminati et al. (1981) recorded the microwave spectrum of CH_3_SC(O)H between 10 and 40 GHz with a cold cell at 235 K. They also performed double resonance microwave measurements that allowed them to confirm the identification of the E-species lines. In total, they measured 22 and 15 lines belonging to the internal A- and E-rotation states, respectively, up to J = 20. They fit the 37 lines using 11 parameters. However, their study shows discrepancies up to 5.7 MHz when comparing the observed line positions to the calculated values (see Tables I and II of the ref. Caminati et al. 1981).

In this context, the goal of the present work is to extend, for the first time, the spectroscopic measurements of CH_3_SC(O)H in the millimeter- and submillimeter-wave spectral ranges in order to produce a reliable line list of frequencies and intensities to be used for searching the molecule in space. Section 2 presents the experimental details, including the synthesis of the S-methyl thioformate. Section 3 contains a presentation of the theoretical model used for data analysis. The second part of this section is dedicated to assignments and fits of the spectral data. Section 4 presents the intensity calculation and the line lists for astronomers. Section 5 corresponds to the astronomical search for CH_3_SC(O)H in the ISM. Section 6 is dedicated to the discussion of our results.

## 2 Experimental details

### 2.1 Chemical synthesis

To prepare S-methyl thioformate CH_3_SC(O)H, we modified the synthesis of Hershfield & Schmir (1972). In a Schlenk flask, methanethiol (methyl mercaptan, CH_3_SH; 0.1 mol) was slowly added to acetic-formic anhydride (0.1 mol) at a temperature ranging from 5 to 10 °C. The flask was immersed in a liquid nitrogen bath, degassed, and then closed with the stopcock. The mixture was stirred for 36 h at 35 °C. Then, a distillation with a short Vigreux Column (20 cm) gave a first fraction containing the S-methyl thioformate and about 30% of formic and acetic acids. Purification was performed in a vacuum line (0.1 mbar) with a first trap cooled at -50 °C to remove both carboxylic acids, and a second trap cooled at -90 °C to selectively condense the S-methyl thioformate, which was thus obtained in a 37% yield.

### 2.2 Spectra measurements

The measurements in the frequency range under investigation (150-660 GHz) were performed using the Lille spectrometer (Zakharenko et al. 2015). The absorption cell was a stainless-steel tube (6 cm in diameter, 220 cm in length). The sample pressure during measurements was about 10 Pa and at room temperature, the line width was limited by Doppler broadening. The frequency ranges, 150-330 and 400-660 GHz, were covered with various active and passive frequency multipliers from Virginia Diodes, Inc.(VDI Inc) and an Agilent synthesizer (12.5-18.25 GHz) was used as the source of radiation. As a detector, we used an InSb liquid He-cooled bolometer from QMC Instruments Ltd to improve the sensitivity of the spectrometer. The sources were frequency modulated at 30 kHz. Estimated uncertainties for measured line frequencies are 30 and 50 kHz depending on the observed signal-to-noise (S/N) and the frequency range. Uncertainties of 100 kHz are also given to relatively broad transitions or presenting a weak S/N.

## 3 Results

### 3.1 Theoretical model

The S-methyl thioformate contains one methyl group internal rotor with a rather low torsional barrier hindering this motion, which was estimated by Caminati et al. (1981) to be V3 = 147.96 cm^–1^. Therefore, the rotational analysis of its spectra requires a suitable theoretical model and Hamiltonian to treat the internal rotation splitting between the A- and E-species.

In the present work, we used the BELGI-Cs^1^ program (Hougen et al. 1994) using the rho-axis method (RAM) with a two-step procedure initially described by Herbst et al. (1984). The BELGI-C_s_ code was previously applied successfully to a number of interstellar molecules containing an internal methyl rotor and was used in particular for the parent species of the cis- methyl formate (Carvajal et al. 2007) as well as for its ^13^C isotopic species H^13^COOCH_3_ and HCOO^13^CH_3_ (Carvajal et al. 2009), ^18^O isotopologues HC^18^OOCH3 and HCO^18^OCH3 (Tercero et al. 2012), and deuterated species DCOOCH3 (Duan et al. 2015). The method takes its name from the choice of the axis system, which allows us to minimize the coupling between internal rotation and global rotation in the Hamiltonian, at least in the zeroth order. As it has been described in the literature (Lin & Swalen 1959; Hougen et al. 1994; Kleiner 2010) and applied extensively, we do not repeat it here.

The BELGI-C_s_ program uses a global approach since it takes into account the A- and E-symmetry states simultaneously, as well as all the **ν*_t_* torsional levels originating from a given vibrational state, up to a truncation level. The interactions between rotation-torsion energy levels are treated explicitly within the Hamiltonian matrix elements. In our approach, only one set of rotational constants and parameters that describe the potential are fit for all the torsional states for a given vibrational state.

In the so-called RAM used in the BELGI-Cs code, we do not use the Principal Axis System (PAS). To simplify the calculations and to get rid at the zeroth order of the torsion-rotation interaction terms, we use a non-principal axis system (called the rho-axis system (RAS); Kleiner 2010). As a consequence, we fit the *D_ab_* parameter, which multiplies the *P_a_P_b_* + *P_b_P_a_* off-diagonal operator, where *P_a_* and *P_b_* designate the projection of the total rotational angular momentum on the *a* and *b* axes, respectively. The *D*_ab_ parameter is related to the θram angle between the *z* axis of the PAS and RAS, as described by Kleiner (2010):

(1) $$\theta_{\text{RAM}}=\frac{1}{2}\arctan\left(\frac{2D_{ab}}{A_{\text{RAM}}-B_{\text{RAM}}}\right)$$

Morever, in the BELGI-C_s_ code, a two-step diagonalisation procedure is used to calculate the energy levels. First, a set of calculations are carried out to diagonalize the torsion part of the Hamiltonian for each K = K_a_ value. Then in the second step, the rest of the Hamiltonian terms (rotational, centrifugal distortion, and interaction terms between rotation and torsion) are diagonalized. The RAM method has been sucessfully applied to a number of astrophysical internal rotors, such as methyl formate (Carvajal et al. 2007, 2009). As described further in Sect. 4, the BELGI-C_s_ program also has an option allowing us to calculate the line strengths (using the RAM values for the electric dipole moments).

The torsional frequency mode *v18* for the cis- S-methyl thioformate is predicted to lie between 80 and 111 cm^–1^ depending on the level of theory used (see Tables III and VII of the ref. Senent et al. 2014). Other low-frequency vibrational modes can also be responsible for rotational transitions observed in the spectrum, such as the *v*_17_ mode (torsion of the CO group) calculated to be around 250 cm^–1^, the v _12_ mode (bending of the CSC bond) observed at 245 cm^–1^ and the v _1_ *1* mode (bending of the OCS bond) observed at 320 cm^–1^ (Senent et al. 2014). However, our present model does not take into account the interactions between the ground state and *v*_17_ *, v*_12_ or *v11.*

### 3.2 Millimeter- and submillimeter-wave spectral analysis

First, we performed a preliminary fit using the microwave data measured by Jones et al. (1976) and Caminati et al. (1981) between 10 and 41 GHz in order to obtain the overall rotation and the internal rotation constants in the RAS using the BELGI-Cs program. These preliminary parameters allowed us to obtain a starting prediction of the millimeter-wave spectrum between 150 and 330 GHz in the ground torsional state **ν*_t_ =* 0. Eventually, we pursued our assignments up to 660 GHz.

Following Jones et al. (1976), the electric dipole moment is maximal along the a principal axis *(*μ _a_ *=* 1.52 D; μ _b_ *=* 0.43 D). Therefore, rotational structures of a-type R-branch series were identified with a shift between 2 and 10 MHz for lines with *K_a_* 2264< 5. Then, lines belonging to higher *J* and *K_a_* values were progressively assigned and added to the fit. After the a-type transitions were analyzed, we also succeeded in assigning some b-type transitions, even though they are approximatively 12 times less intense $\left[{\left(\frac{\mu_{a}}{\mu_{b}}\right)}^{2}\approx 12.5\right]$. In this process, we simultaneously assigned A- and E-type transitions, for *J* values up to 75. The same assignment process was pursued in the first and second excited torsional states *ν*_t_ = 1 and 2.

**Fig. 1:**
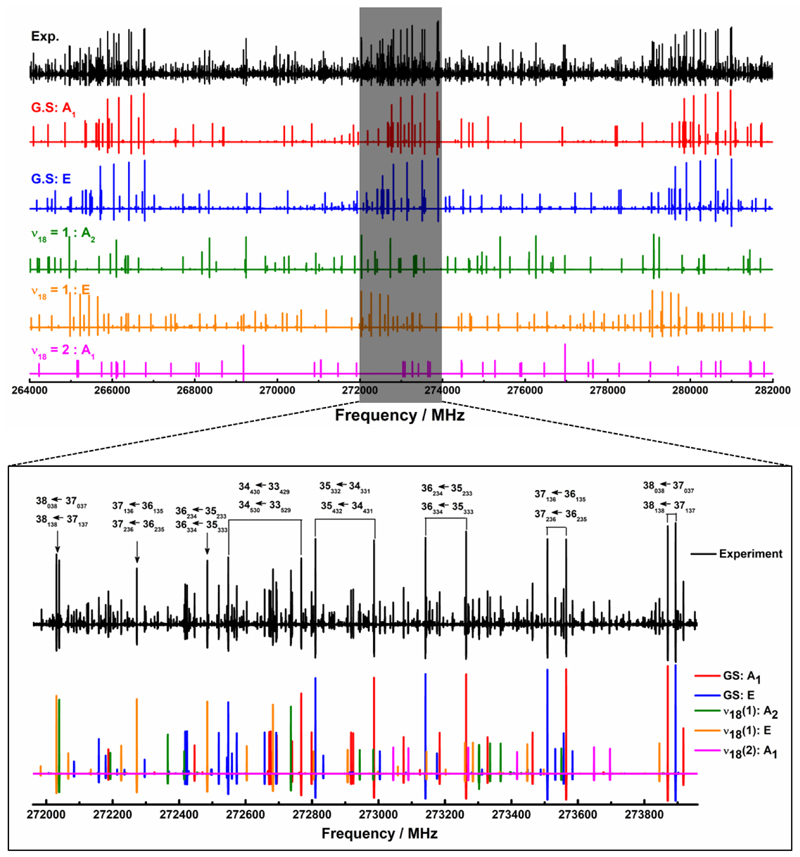
*Upper panel* : section of experimental spectrum between 264 and 282 GHz (in black) compared to calculated spectra obtained from our fit parameters described in Table 2. *Lower panel* : expanded view of the 271.96-273.96 GHz region showing the assignments (A-species of the GS in red, E-species of the GS in blue, A-species of the *ν*_18_ =1 mode in green, E-species of the *ν*_18_ =1 mode in orange, and A-species of the *ν*_18_ =2 mode in magenta).

The spectrum was recorded at room temperature close to 300 K. For this reason, it was very dense (as shown in Fig. 1) since it contains not only rotational transitions of the ground state but also those belonging to the low-lying excited states, as mentioned earlier in Sect. 3.1.

In total, 5154 rotational transitions were assigned in our model and fit with experimental accuracy for *J* and *K_a_* values up to 75 and 24, respectively. We weighted all lines into four sets corresponding to differences in measurement accuracy. The first set corresponds to isolated lines with a weight corresponding to $\frac{1}{{\left(\Delta \nu \right)}^{2}}$, where ∆*ν* is the measurement uncertainty of 30 kHz. The second set is weighted corresponding to the measurement accuracy of 50 kHz since they are blended lines and their energy levels are degenerated. The third given set weighted to 100 kHz corresponds to relatively broad transitions or those with a weak (S/N) ratio. Finally, we weighted the microwave transitions measured in earlier studies by Jones et al. (1976) and Caminati et al. (1981) to 150 kHz. Indeed, in our fit those lines seem to fit with larger residuals than the measurement accuracy of 100 kHz estimated by those authors. The quality of our fit is presented in Table 1, with the unitless standard deviations for each torsional state.

A global unitless standard deviation of 1.01 was obtained by floating 61 parameters for 3599 transitions belonging to the ground state *ν*_t_ = 0, 1391 transitions for *ν*_1_ *8 =* 1, and 164 transitions for *ν*_1_ *8 = 2.* Table 2 shows the values of the 61 molecular parameters obtained by the fit using the BELGI-C_s_ code together with the operators that those parameters multiply. The three rotational constants *A, B,* and *C* as well as the quartic centrifugal distortion *(∆_J_, ∆_JK_, ∆_K_, δ_J_ and δ_K_)* and three sextic constants (H_J_, H_KJ_ and H_K_) were determined. As explained in Sect. 3.1, in the RAM we also need to fit a parameter (D_ab_) that corresponds to the fact that the inertial tensor is not diagonal. The height of the potential barrier V_3_ was determined as 127.4846(15) cm^–1^. We note that as we globally fit the data from *ν*_t_ = 0, 1 and 2, we were also able to determine the value of the internal rotation constant F = 5.332339(21) cm^–1^, and the second and the third terms in the cosine expansion of the potential (V_6_ = 24.3418(94) cm^–1^, V_9_ = 6.567(21) cm^–1^). Forty-five interaction parameters between rotation and torsion (such as ρ, c_2_, c_4_, k_5_, k_2_, k_1_, etc.), as defined in Table 2, were also necessary to reproduce the observed transitions.

Using the set of parameters of Table 2, the millimeter- and submillimeter-wave spectra measured between 150 and 660 GHz are very well reproduced to experimental accuracies, and the main strong rotational lines are assigned as shown in Fig. 1, where a section of 18 GHz (upper part) is presented with a zoomed portion of 2 GHz (lower part).

**Fig. 2:**
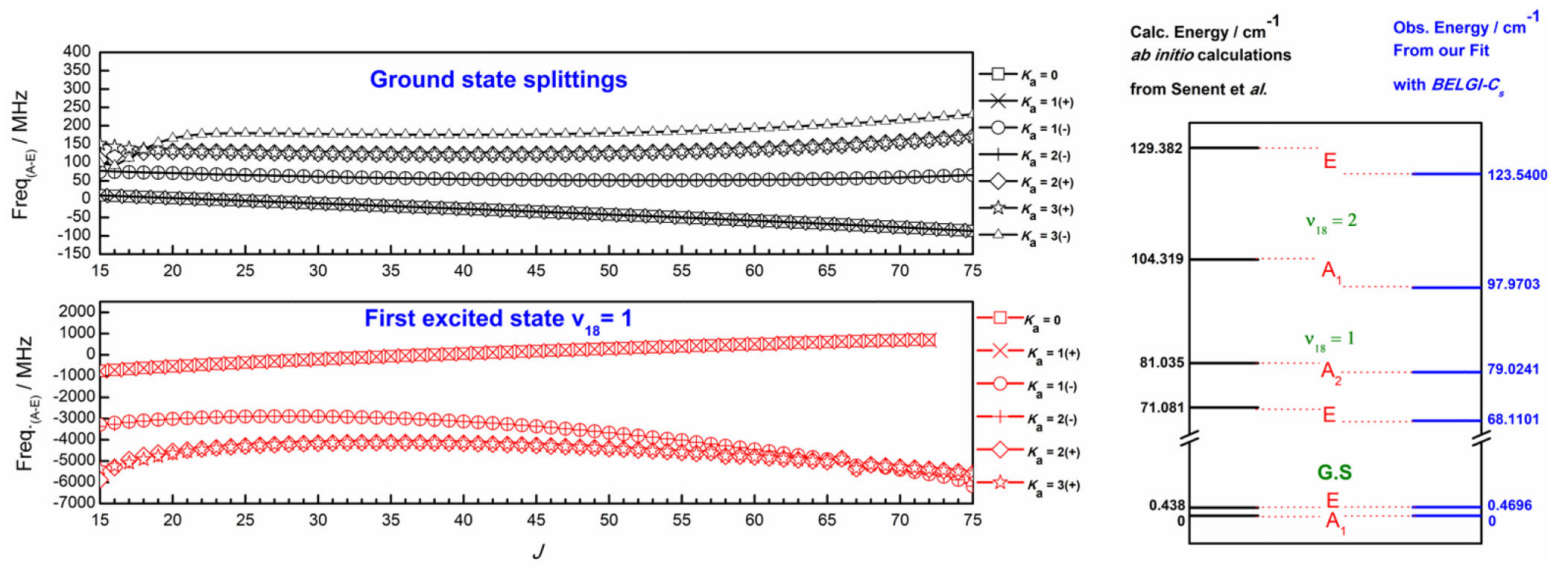
*Left plot* : variation of A-E splittings with J values for R-type branches of CH_3_SC(O)H in the ground state (upper trace) and in the first torsional state *ν*_18_ = 1 (lower trace). *Right plot* : obtained energy levels from our BELGI-C_s_ analysis compared to those determined by Senent et al. (2014) with quantum chemical calculations.

The internal rotation effect for the CH3SC(O)H molecule is quite large in comparison with methyl formate. Its barrier height V3 is determined to be relatively low in comparison with its oxygenated analog methyl formate, CH3OC(O)H, for which V3 = 372.6720(42) cm^–1^ (Ilyushin et al. 2009). As a result, the A-E splittings are larger in our case where, for *K_a_* < 3, differences between A- and E-frequencies vary from -150 to 250 MHz instead of only few MHz for methyl formate for the ground torsional *ν*_t_ *=* 0 state. Moreover, this splitting between A-and E-levels becomes larger for excited torsional states (*ν*_1_8 = 1 and *ν*_1_8 = 2), going up to some GHz. The left panel of Fig. 2 shows the variation of those A-E splittings with increasing *J* for ground state (upper trace) and for the first excited state (lower trace). Therefore, the use of a suitable theoretical model based on the RAM is needed to predict these rotational lines correctly. Indeed, the global approach used in the BELGI-C_s_ code allows us to treat all the torsional states together in the fit and to deal with the torsion-rotation interaction terms.

In the right panel of Fig. 2, we also compare the torsional energy levels determined by ab initio calculations (Senent et al. 2014) with those determined using our parameters from the fit. The agreement between our observations and those calculations is quite good.

## 4 Intensity calculation

For calculations of the line strengths, we used the same procedure as the one described in detail in Sect. 3.2 of Kleiner 2010. This method had been used for different molecules with C_s_ symmetry containing one methyl rotor such as methyl formate and its isotopologues (Favre et al. 2014; Carvajal et al. 2009, 2007; Haykal et al. 2014; Tercero et al. 2012; Duan et al. 2015) or vinyl acetate (Kolesnikova et al. 2015). We used the BELGI-C_s_ code to calculate the line strengths, using the energy parameters determined in Table 2. In the same way that the Hamiltonian was used in the calculation and fit of the line positions in the RAM, the calculation of the line strengths also has to be carried out in this same axis system. For this purpose, the components of the dipole moment μ_a_ and μ_b_ obtained by Stark measurements (Jones et al. 1976) were thus transformed in the RAM system using Eq. (20) of Kleiner (2010) and determined to be 1.527 Debye and -0.404 Debye, respectively. The value of the angle between the PAS and the RAS was calculated using Eq. (1) to be 30.6°.

The line frequencies, line strengths (S.μ^2^), and lower state energies, as well as the assignments for the observed transitions of CH_3_SC(O)H in the millimeter-wave and submillimeter-wave ranges are shown in Table A.1. Using a method and a code developed by Favre et al. (2014); Carvajal et al. (2019), we calculated the partition function for an internal rotor, including the torsional contribution. The values of the partition function are presented in Table A.2 for temperatures allowing the search of this molecule in different regions in warm molecular clouds of the ISM.

## 5 Search for methyl thioformate in space

Using the rotational constants of Table 2 and the MADEX code (Cernicharo 2012), we searched for methyl thioformate in warm molecular clouds. High-mass star-forming regions are the best candidates for this search. On one hand, it is well known that methyl formate is one of the most abundant species in hot cores. In addition, these regions are associated with molecular outflows in which large abundances of sulfur-bearing species arise (see e.g., Esplugues et al. 2013; Crockett et al. 2014; Luo et al. 2019; Belloche et al. 2013). On the other hand, one of the HCOOCH_3_ derivatives, ethyl formate (HCOOCH_2_CH_3_), has only been unambiguously detected in this kind of region (Belloche et al. 2009, 2013; Tercero et al. 2013, 2015, 2018; Rivilla et al. 2017; Marcelino et al. 2018). Therefore, we mainly focused on the available astronomical data of Orion KL and Sgr B2. Our frequency predictions are sufficiently reliable for a-type transitions up to 300 GHz, which is the range of the astronomical search. Nevertheless, owing to the high line density of the data and in order to ensure the line identification, we restricted our search to transitions with uncertainties in the predicted frequency of less than 0.1 MHz.

We used the ALMA science verification (SV) data of Orion KL between 213.7 and 246.6 GHz (for observations and data reduction, see e.g., Tercero et al. 2018) and the IRAM 30 m, 3 mm survey of Sgr B2 provided by Belloche et al. (2013). In the ALMA data, we concentrated the search in the emission peaks of methyl (MF and ET peaks of Tercero et al. 2018). These positions also seem to be associated with the molecular outflow driven by source I (see Tercero et al. 2018 and references therein).

We did not find this species above the detection limit of both sets of data. To provide upper limits to its column density, we used MADEX to derive the synthetic spectrum of CH_3_SC(O)H for both sources assuming local thermodynamic equilibrium (LTE) and the same physical parameters as those derived for methyl formate by Tercero et al. (2018) and Belloche et al. (2013) in Orion KL and Sgr B2, respectively. The results are summarized in Table 3.

These results provide an abundance ratio of $\frac{\left[{\text{HCOOCH}}_{\text{3}}\right]}{\left[{\text{CH}}_{\text{3}}\text{SC}\left(\text{O}\right)\text{H}\right]}>10^{3}$ in the emission peaks of methyl formate within Orion KL (Tercero et al. 2018) and a value >14-20 for the different methyl formate components of Sgr B2 (Belloche et al. 2013). It is worth noting that the latter value may be greatly underestimated due to the high level of line confusion in the IRAM 30m data of Sgr B2, which leads to a less constrained upper limit for the CH_3_SC(O)H column density in this region.

## 6 Discussion

The values for the rotational constants A, B, and C with the BELGI-C_s_ code are obtained via the RAM. To compare them directly with the values derived from the previous microwave study (Caminati et al. 1981) or with the values calculated by ab initio methods (Senent et al. 2014), we need to transform them from the RAS to the PAS. This transformation is done with a rotation by an angle θ_RAM_ between the z axis of both systems, as described in Sect. 3.1 using Eq. (1) (Kleiner 2010).

Table 4 shows the values of the rotational constants after transforming back to the PAS and comparing with those determined in the literature. The spectroscopic parameters A, B, and C carried out in the present study are more accurate, but they are slightly different from those determined in the previous experimental microwave investigations (Caminati et al. 1981). They also differ from the values determined by previous theoretical calculations (Senent et al. 2014) by 0.5–1%. This could mainly be explained by the fact that we used a different Hamiltonian based on a “global approach”, taking into account both ground state and excited torsional states with a determination of higher order interaction terms between overall and internal rotations (shown in Table 2).

**Fig. 3:**
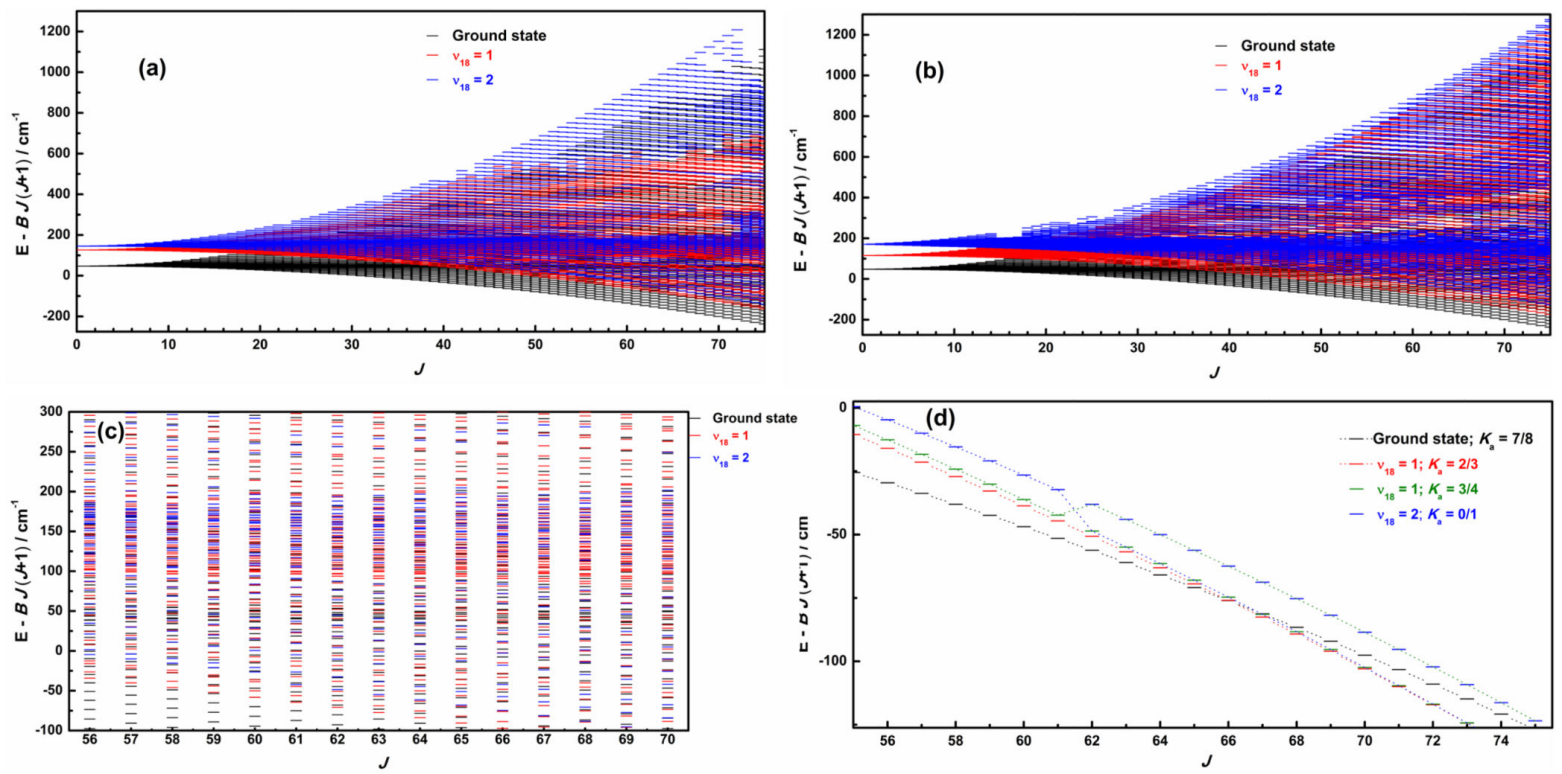
*(a,b)* Torsion-rotation diagrams (reduced energy) [*E - BJ(J + 1)*] in the ground state, the *ν*_18_ = 1 and *ν*_18_ = 2 states of torsion for both A-species (*left plot*) and E-species (*right plot*). (c) An enlargement of the torsion-rotation diagram for J values between 56 and 70 in order to highlight how energy levels become close to each other. (d) Examples of perturbations between different torsional states (avoided crossings are shown around *J* = 61 and 62, as well as around *J* = 66).

In Fig. 3, we show the torsion-rotation interaction diagrams, which represent the reduced energy levels *(E - BJ(J* + 1)) in terms of the rotational quantum number *J.* As shown in Figs. 3a and b for A- and E-species, respectively, the torsional levels in ground state with J ≥ 16 begin to be mixed, though rotation-torsional interactions or resonances, with those of *ν*_18_ = 1 and 2 excited states. Figure 3c presents a zoomed section of Fig. 3a to highlight the complexity of the interaction scheme for *J* ≥ 56. As shown in Fig. 3d, the interactions often correspond to the rotation-torsion states’ avoided crossings (with large differences in the *K* values between the interacting energy levels), such as between *ν*_t_ = 0, for values of *K_a_ =* 7 and *ν*_18_ = 1 for 2 ≤ *K_a_* ≤ 4, and *ν*_18_ = 2, *K_a_ ≤* 1.

Our global fit represents a clear improvement on previous work, especially for the ground torsional state and the first excited state, which are fit with 54 and 120 kHz standard deviations, respectively. However our analysis only contains a limited number of rotational transitions within *ν*_1_ *8=2,* with 164 transitions belonging to the A-species. No E-species could be assigned without ambiguity for this state. Presumably the rotational excited torsional states of S-methyl thioformate interact with the levels from other low-frequency vibrational modes, such as the bending of the CSC bond *ν*_1_2(a’) observed at 245 cm^–1^ (Jones et al. 1976) and the torsional mode *ν*_17_ of the CO calculated at 253 cm^–1^ (Senent et al. 2014). A natural next step in the understanding of the rotation-vibrational-torsional spectrum of S-methyl thioformate will be the recording of the far-infrared spectrum at high resolution, in order to measure the torsional fundamental bands at low frequencies. As in the case of methyl formate, the assignment and fit of the higher excited torsional states of S-methyl thioformate will also depend on the global treatment of the various vibrations (Ilyushin et al. 2009). It is possible that the *ν*_18_ *= 2* and *ν*_1_ *8 =* 3 analysis will require significant extension of the current model to include suspicious perturbations from the *ν*_12_ bending mode at 245 cm^–1^, or **ν**_17_ torsion mode at 253 cm^–1^.

## 7 Conclusion

This work presents a global fit consisting of pure rotational transitions and rotation-torsion transitions using the BELGI-C_s_ code for an internal rotor. The present fit makes it possible to reproduce, from microwave to submillimeter-wave domains, data up to 660 GHz with root mean square deviations close to experimental accuracies. Using our spectroscopy work, a deep search of S-methyl thioformate was carried out in the IRAM 30 m and ALMA data of different high-mass star-forming regions (Orion KL and Sgr B2). We derive an upper limit to the CH_3_SC(O)H column density in these regions.

## Supplementary Material

Appendix

## Figures and Tables

**Table 1:** Overview on the quality of measured lines included in the BELGI-C_s_ fit, for the ground state (GS) and the two first excited states *ν*_18_ =1 and *ν*_18_ = 2.

	Mm-wave and submm-wave data			Mw data^(a)^
	G.S	*ν*_18_ =1	*ν*_18_ =2	G.S
*J_max_*	75	75	75	20
*K*_max_	24	13	3	6
*N_A_/N_E_^(b)^*	1831/1714	720/671	164/0	43/11
Std. dev.^(c)^	0.89	1.20	1.43	0.66

(a) **Notes** These 54 lines were measured by Jones et al. (1976) and Caminati et al. (1981) in the microwave domain between 10 and 41 GHz, and they were added to our new measured 3545 rotational transitions in the final fit. The total number of ground-state *ν*_t_ = 0 transitions is thus 3599.

(b) Number of A- and E-transitions included in our BELGI-C_s_ global fit.

(c) Unitless standard deviation of the fit.

**Table 2:** Spectroscopic parameters of CH_3_SC(O)H in the RAM obtained with the BELGI-C_s_ program.

*nlm^(a)^*	Operator^*(b)*^	Par.^*(c)*^	Value^*(d)*^	nlm^*(a)*^	Operator^*(b)*^	Par.^*(c)*^	Value^*(d)*^
220	$\frac{1}{2}(1-\cos3\alpha )$	*V_3_*	127.4846(15)				
	$P_{\alpha }^{2}$	*F*	5.332339(21)		(1 – cos 6α)(*P_a_P_b_ + P_b_P_a_*)	*dd_ab_*	3.4755(32) × 10^–4^
211	*P_α_P_a_*	*ρ*	0.03644877(22)		$\left(1-\cos6\alpha \right)\left(P_{b}^{2}-P_{c}^{2}\right)$	*c*_11_	4.376(19) × 10^–5^
202	$P_{a}^{2}$	*A*	0.22208287(97)		$P_{\alpha }^{4}P_{a}^{2}$	*K_1_*	3.546(18) × 10^–7^
	$P_{b}^{2}$	*B*	0.3162569(10)		$P_{\alpha }^{4}P^{2}$	*M_v_*	2.519(39) × 10^–8^
	$P_{c}^{2}$	*C*	0.119194004(55)		(1 – cos 6α)$P_{a}^{2}$	*K*_2_	2.2583(42) × 10^–4^
	*(P_a_P_b_ + P_b_P_a_)*	*D_ab_*	0.08605178(54)	624	(1 – cos 3α)P^2^ $P_{a}^{2}$	*k_5J_*	7.098(84) × 10^–8^
440	$\frac{1}{2}(1-\cos6\alpha )$	*V*_6_	24.3418(94)		(1 – cos 3α)$P_{a}^{4}$	*k_5K_*	7.145(86) × 10^–8^
	$P_{\alpha }^{4}$	*k*_4_	-9.832(68) × 10^–4^		(1 – cos 3α)P^4^	*f_v_*	8.330(78) × 10^–9^
431	$P_{\alpha }^{3}$ *P_a_*	*k*_3_	1.2310(35) × 10^–4^		(1 – cos 3α)($P_{b}^{2}$ – $P_{c}^{2}$)P^2^	*c_2J_*	4.613(78) × 10^–9^
	$P_{\alpha }^{3}$ *P*_b_	*ESPOIR*	-5.134(32) × 10^–5^		(1 – cos 3α){$P_{a}^{2}$,($P_{b}^{2}$ – $P_{c}^{2}$)}	*c_2K_*	5.023(27) × 10^–8^
422	$P_{\alpha }^{2}$P^2^	*G_v_*	-9.521(32) × 10^–6^		(1 – cos 3α)(P_a_P_b_ + P_b_P_a_)P^2^	*d_abJ_*	2.639(40) × 10^–8^
	2$P_{\alpha }^{2}$($P_{b}^{2}$ – $P_{c}^{2}$)	*c*_1_	-5.088(17) × 10^–6^		(1 – cos 3α)($P_{a}^{3}$ *P_b_ + P_b_* $P_{a}^{3}$)	*d_abK_*	5.188(44) × 10^–8^
	(1 – cos 3α)P^2^	*F_v_*	-8.7206(42) × 10^–4^		$P_{\alpha }^{2}$ $P_{a}^{2}$P^2^	*k_2J_*	9.19(45)× 10^–11^
	(1 – cos 3α)$P_{a}^{2}$	*k*_5_	1.4439(14) × 10^–3^		*sin3αP^2^{P_a_, P_c_}*	*D_acJ_*	4.98(26) × 10^–9^
	(1 – cos 3α)($P_{b}^{2}$ – $P_{c}^{2}$)	*c*_2_	2.3162(44) × 10^–4^		*sin3αP^2^{P_b_, P_c_}*	*D_bcJ_*	3.028(26) × 10^–9^
	(1 – cos 3α)(*P_a_P_b_* + *P_b_P_a_*)	*d_ab_*	-3.47891(53) × 10^–3^	633	$P_{\alpha }^{3}$ *P^2^P_a_*	*k3J*	3.18(17) × 10^–9^
	$P_{\alpha }^{2}$ $P_{a}^{2}$	*k*_2_	7.797(87) × 10^–6^		$P_{\alpha }^{3}$ $P_{a}^{3}$	*k_3K_*	1.731(29) × 10^–8^
	$P_{\alpha }^{2}$ *(P_a_P_b_ + P_b_P_a_)*	*∆_ab_*	5.640(27) × 10^–6^		*P_a_$P_{\alpha }^{3}$ ($P_{b}^{2}$ – $P_{c}^{2}$)*	*c_12_*	1.287(84) × 10^–9^
	*sin 3α(P_a_P_c_ + P_c_P_a_)*	*D_ac_*	5.47(15) × 10^–5^	606	*P* ^6^	*H_J_*	0.02220(10) × 10^–12^
	*sin 3α(P_b_P_c_ + P_c_P_b_)*	*D_bc_*	1.320(12) × 10^–4^		*P^2^* $P_{a}^{4}$	*H_kj_*	2.112(16)× 10^–12^
413	*P_α_P_a_P^2^*	*L_v_*	3.4869(48) × 10^–6^		$P_{a}^{6}$	*H_K_*	2.854(27)× 10^–12^
	*P_α_* $P_{a}^{3}$	*k*_1_	-3.0770(63) × 10^–6^	660	*½ (1 – cos 9α)*	*V*_9_	6.567(21)
	*P_α_{P_a_, ($P_{b}^{2}$ – $P_{c}^{2}$)}*	*c*_4_	1.8914(18) × 10^–6^		$P_{\alpha }^{6}$	*k_4B_*	1.946(26) × 10^–5^
	*P_a_P_α_(P_a_P_b_ + P_b_P_a_)*	*δ_ab_*	-1.7256(37) × 10^–6^	651	$P_{\alpha }^{5}$ *P_a_*	*k_3B_*	4.450(17) × 10^–6^
404	*-P^4^*	*∆_J_*	1.9298(55) × 10^–7^	615	*P_a_P_α_P^2^* $P_{a}^{2}$	*k_1J_*	3.776(69) × 10^–11^
	*-P^2^* $P_{a}^{2}$	*∆_JK_*	3.590(18) × 10^–7^		*P_a_P_α_* $P_{a}^{4}$	*k_1K_*	5.12(11)× 10^–11^
	-$P_{a}^{4}$	*∆_K_*	-5.3604(43) × 10^–7^		*P_a_P_α_{$P_{a}^{2}$, ($P_{b}^{2}$ – $P_{c}^{2}$)}*	*c_4K_*	6.54(25) × 10^–12^
	*-2P^2^($P_{b}^{2}$ – $P_{c}^{2}$)*	*δ_J_*	0.7803(27) × 10^–7^	844	(1 – cos 6α)P^4^	*N_vJ_*	6.710(16)× 10^–10^
	*-{$P_{a}^{2}$,(P^2^ – $P_{c}^{2}$)}*	*δ_K_*	2.6080(13) × 10^–7^		(1 – cos 6α)P^2^ $P_{a}^{2}$	*K_2j_*	2.445(63) × 10^–9^
	(*P_a_P_b_* + *P_b_P_a_*)*P* ^2^	*D_abJ_*	9.768(58) × 10^–8^				
642	(1 – cos 6α)*P* ^2^	*N_v_*	-1.6163(20) × 10^–4^				

Notes. All values of these rotational constants are in cm^–1^, except for ρ, which is unitless.

(a) n = l + m, where n is the total order of the operator, l is the order of the torsional part, and m is the order of the rotational part.

(b) P_a_, P_b_ and P_c_ are the components of the overall rotational angular momentum. P_α_ is the angular momentum of the internal rotor. {u,v} is the anticommutator uv+vu.

(c) The product of the parameter and operator from a given row yields the term actually used in the vibration-rotation-torsion Hamiltonian, except for F, ρ and A, which occur in the Hamiltonian in the form F(P_α_ – ρP_a_)^2^ + AP_a_

(c) Values of the parameters from the present fit for *ν*_t_ = 0, 1, and 2. Statistical uncertainties are given in parentheses in units of the last quoted digits.

**Table 3:** S-methyl thioformate column densities.

	Orion KL^(a)^ (MF peak)	Orion KL^(a)^ (ET peak)	Sgr B2^(b)^ (IRAM 30m)
Coordinates	α= 05^h^ 35^m^ 14.1^s^	α =05^h^ 35^m^ 14.4^s^	α = 17^h^47^m^20.0^s^
(J2000.0)	*δ* = -05° 22′ 36.8″	*δ* = -05° 22′ 34.74″	δ = -28°22′19.0″
*HPBW* ^(c)^ (″)	~2.0×1.5	~2.0×1.5	30-21
Freq.^(d)^ (GHz)	213.7-246.6	213.7-246.6	80-115.5
υ_LSR_ (km s^–1^)	7.5	8.0	63.5/ 73.5
∆υ_FWHM_ (km s^–1^)	2.0	3.0	7.0 / 7.0
*d_sou_* (″)	3.0	3.0	4.0 / 4.0
T_rot_ (K)	150	150	80/ 80
N(CH_3_SCOH) (cm^–2^)	≤1.0x 10^14^	≤1.0x 10^14^	≤2.0/1.0x 10

(a) **Notes.** Physical parameters derived for the main spectral component of HCOOCH_3_ by Tercero et al. (2018).

(b) Physical parameters derived for two spectral components of HCOOCH_3_ by Belloche et al. (2013).

(c) Half power beam width (HPBW) for observations with a single-dish telescope (IRAM 30m) and synthetic beam for the ALMA SV observations.

(d) Range of frequencies considered in the analysis.

**Table 4:** Comparison of spectroscopic constants of CH_3_SC(O)H, obtained by BELGI-C_s,_ fit with those determined in previous experimental and theoretical investigations.

Parameter	Unit	Present work	Previous Mw work^(a)^	Ab initio study^(b)^	
				Ground state	*ν*_18_ =1
A	MHz	11 010.242(32)	11 035.13(19)	11043.16	11 117.07
B	MHz	5128.785(88)	5099.99(13)	5113.37	5061.61
C	MHz	3573.3379(28)	3561.570(30)	3570.06	3552.91
∆_J_	kHz	5.785301(16)	3.10(50)	3.482	
V_3_	cm^–1^	127.4846(15)	150.47(1.67)	139.7	
V_6_	cm^–1^	24.3418(94)		25.4	
N^(c)^		5154	37		
Std. dev.	unitless	1.01			

**Notes.** Overall rotational constants *A*, *B*, and *C* are transformed into the PAS, as explained in the text.

(a) Constants taken from the previous microwave study (*ν*_t_ = 0) of Caminati et al. (1981).

(b) Values obtained by Senent et al. (2014) using ab initio calculations at the CCSD(T)/VTZ level of theory.

(c) Total number of measured lines used in the fit.

## Footnotes

1 The source code BELGI for the fit, an example of input data file and a readme file are available at the web site PROSPE, managed by Dr. Zbigniew Kisiel. Extended versions of code are also available by contacting the author I. Kleiner.
