# Supplementary material for: Rotational spectroscopic study of S-methyl thioformate: A global laboratory analysis of ground and excited torsional states up to 660 GHz
Source: Astron Astrophys. Author manuscript; Available in PMC 2021 Feb 15. (PMC7116753; doi:10.1051/0004-6361/202038200)
Supplement: Appendix [file EMS114945-supplement-Appendix.pdf]

Appendix A: Additional tables

Table A.1. Excerpt of the line list for $CH_3SC(O)H$ in the microwave and millimeter-wave range.

Upper state				Lower state										
v_t	J	K_a	K_c	v_t	J	K_a	K_c	Sym.	Obs. MHz	Calc. MHz	Obs. – Calc. MHz	E_{up} cm^{-1}	E_{low} cm^{-1}	$S*\mu^2$ $Debye^2$
0	2	1	2	0	1	1	1	A	15 804.360(150)	15 804.262	0.098	48.2813	47.7541	0.192588E+01
0	2	1	2	0	1	1	1	E	15 807.960(150)	15 808.067	−0.107	48.7485	48.2212	0.189151E+01
0	2	0	2	0	1	0	1	E	17 003.290(150)	17 003.268	0.022	48.5911	48.0239	0.250867E+01
0	2	0	2	0	1	0	1	A	17 092.040(150)	17 092.095	−0.055	48.1266	47.5565	0.254265E+01
0	16	5	11	0	15	5	10	E	150 541.933(30)	150 541.964	−0.031	94.3548	89.3333	0.186967E+02
0	16	5	11	0	15	5	10	A	151 336.356(30)	151 336.343	0.013	94.0586	89.0106	0.187751E+02
1	21	0	21	1	20	0	20	A	151 567.769(100)	151 567.960	−0.191	182.6137	177.5580	0.230444E+02
1	21	1	21	1	20	1	20	A	151 567.769(100)	151 567.483	0.286	182.6137	177.5580	0.230444E+02
1	21	1	21	1	20	1	20	E	152 072.790(100)	152 072.771	0.019	172.0147	166.9421	0.285556E+02
1	21	0	21	1	20	0	20	E	152 072.790(100)	152 072.789	0.001	172.0147	166.9421	0.285556E+02
2	24	1	23	2	23	1	22	A	183 932.733(50)	183 932.639	0.094	226.8075	220.6722	0.327233E+02
0	21	20	1	0	20	20	0	A	183 952.286(50)	183 952.242	0.044	203.5658	197.4298	0.250842E+01
0	21	20	2	0	20	20	1	A	183 952.286(50)	183 952.242	0.044	203.5658	197.4298	0.250842E+01
2	25	0	25	2	24	0	24	A	184 013.640(50)	184 013.698	−0.058	225.8189	219.6808	0.319334E+02
1	28	0	28	1	27	0	27	A	201 218.098(100)	201 218.032	0.066	224.6298	217.9179	0.369771E+02
1	28	1	28	1	27	1	27	A	201 218.098(100)	201 218.032	0.066	224.6298	217.9179	0.369771E+02
0	23	6	18	0	22	6	17	E	201 240.369(30)	201 240.379	−0.010	137.3375	130.6249	0.270077E+02
0	23	22	2	0	22	22	1	E	201 325.474(30)	201 325.440	0.034	235.4292	228.7137	0.250758E+01
0	23	22	1	0	22	22	0	A	201 389.321(50)	201 389.268	0.053	235.3366	228.6190	0.248480E+01
0	23	22	2	0	22	22	1	A	201 389.321(50)	201 389.268	0.053	235.3366	228.6190	0.248480E+01
1	28	1	28	1	27	1	27	E	201 487.949(100)	201 487.963	−0.014	214.1165	207.3955	0.663354E+02
1	28	0	28	1	27	0	27	E	201 487.949(100)	201 487.963	−0.014	214.1165	207.3955	0.663354E+02
0	39	2	37	0	38	2	36	E	294 442.233(50)	294 442.247	−0.014	254.2485	244.4269	0.815427E+02
0	39	3	37	0	38	3	36	E	294 442.233(50)	294 442.247	−0.014	254.2485	244.4269	0.815427E+02
0	40	2	38	0	39	2	37	A	301 662.222(50)	301 662.212	0.010	264.0225	253.9602	0.589426E+02
0	40	3	38	0	39	3	37	A	301 662.222(50)	301 662.212	0.010	264.0225	253.9602	0.589426E+02
1	65	1	65	1	64	1	64	E	461 957.557(100)	461 957.616	−0.059	628.0309	612.6216	0.150728E+03
1	65	0	65	1	64	0	64	E	461 957.557(100)	461 957.616	−0.059	628.0309	612.6216	0.150728E+03
0	75	1	74	0	74	1	73	A	542 497.712(50)	542 497.777	−0.065	748.5724	730.4766	0.182046E+03
0	75	2	74	0	74	2	73	A	542 497.712(50)	542 497.777	−0.065	748.5724	730.4766	0.182046E+03
2	75	1	74	2	74	1	73	A	544 005.163(50)	544 004.966	0.197	849.2223	831.0762	0.174155E+03
0	75	5	70	0	74	5	69	E	568 540.980(50)	568 540.717	0.263	812.0658	793.1013	0.152061E+03
0	75	6	70	0	74	6	69	E	568 540.980(50)	568 540.718	0.262	812.0658	793.1013	0.152061E+03
0	74	6	68	0	73	6	67	A	568 684.774(100)	568 684.805	−0.031	808.1602	789.1909	0.156142E+03
0	74	7	68	0	73	7	67	A	568 684.774(100)	568 684.806	−0.032	808.1602	789.1909	0.156142E+03
0	75	5	70	0	74	5	69	A	569 084.432(50)	569 084.411	0.021	812.2936	793.3110	0.162207E+03
0	75	6	70	0	74	6	69	A	569 084.432(50)	569 084.411	0.021	812.2936	793.3110	0.162207E+03

Notes. The table includes assignments, observed frequencies, calculated frequencies from the *BELGI* – C_s fit, residuals, upper- and lower-state energy levels, and line strengths $S*\mu^2$. The complete table is available at the CDS.

Table A.2. Partition functions of S-methyl thioformate, calculated at different temperatures.

T (K)	Q_{rot} $^{(a)}$	Q_{tor} $^{(b)}$	Q_{vib} $^{(c)}$	Q_{RTV} $^{(d)}$
80.0	8501.8	2.83	1.03	24 781.9
150.0	21 828.0	3.62	1.27	100 352.0
225.0	40 100.6	4.18	1.85	310 097.9
300.0	61 738.9	4.53	2.86	799 876.8

Notes. The partition function is calculated using the method described in Carvajal et al. (2019). The experimental vibrational energies are taken from Jones et al. (1976), and the torsional band ν_{17} (CO-torsion) is calculated with CCSD(T) by Senent et al. (2014). The nuclear spin degeneracy is assumed as 1. $^{(a)}$Classical rotational partition function, calculated for the A-species (the same value is expected for the E-species within this approximation) using rotational constants from Jones et al. (1976). $^{(b)}$The torsional partition function, calculated using v_t = 0, 1, and 2. $^{(c)}$The vibrational partition function, calculated using all the fundamental energy levels except the CH_3 torsional mode. $^{(d)}$The rotation-torsion-vibration partition function.
